# Diagnostic value of exome and whole genome sequencing in craniosynostosis

**DOI:** 10.1136/jmedgenet-2016-104215

**Published:** 2016-11-24

**Authors:** Kerry A Miller, Stephen R F Twigg, Simon J McGowan, Julie M Phipps, Aimée L Fenwick, David Johnson, Steven A Wall, Peter Noons, Katie E M Rees, Elizabeth A Tidey, Judith Craft, John Taylor, Jenny C Taylor, Jacqueline A C Goos, Sigrid M A Swagemakers, Irene M J Mathijssen, Peter J van der Spek, Helen Lord, Tracy Lester, Noina Abid, Deirdre Cilliers, Jane A Hurst, Jenny E V Morton, Elizabeth Sweeney, Astrid Weber, Louise C Wilson, Andrew O M Wilkie

**Affiliations:** 1 Clinical Genetics Group, Weatherall Institute of Molecular Medicine, University of Oxford, Oxford, UK; 2 Computational Biology Research Group, Weatherall Institute of Molecular Medicine, University of Oxford, Oxford, UK; 3 Department of Clinical Genetics, Oxford University Hospitals NHS Foundation Trust, Oxford, UK; 4 Craniofacial Unit, Oxford University Hospitals NHS Foundation Trust, Oxford, UK; 5 Department of Craniofacial Surgery, Birmingham Children's Hospital NHS Foundation Trust, Birmingham, UK; 6 North East Thames Regional Genetics Service, Great Ormond Street Hospital for Children NHS Foundation Trust, London, UK; 7 Oxford Medical Genetics Laboratories, Oxford University Hospitals NHS Foundation Trust, Oxford, UK; 8 Oxford Biomedical Research Centre, National Institute for Health Research, Oxford, UK; 9 Wellcome Trust Centre for Human Genetics, University of Oxford, Oxford, UK; 10 Department of Plastic and Reconstructive Surgery and Hand Surgery, Erasmus Medical Centre, University Medical Centre Rotterdam, Rotterdam, The Netherlands; 11 Department of Bioinformatics, Erasmus Medical Centre, University Medical Centre Rotterdam, Rotterdam, The Netherlands; 12 Department of Paediatric Endocrinology, The Royal Belfast Hospital for Sick Children, Belfast, UK; 13 Clinical Genetics Unit, Birmingham Women's Hospital NHS Foundation Trust, Birmingham, UK; 14 Department of Clinical Genetics, Liverpool Women's NHS Foundation Trust, Liverpool, UK

**Keywords:** Craniosynostosis, Exome/whole genome sequencing, Actionable mutation

## Abstract

**Background:**

Craniosynostosis, the premature fusion of one or more cranial sutures, occurs in ∼1 in 2250 births, either in isolation or as part of a syndrome. Mutations in at least 57 genes have been associated with craniosynostosis, but only a minority of these are included in routine laboratory genetic testing.

**Methods:**

We used exome or whole genome sequencing to seek a genetic cause in a cohort of 40 subjects with craniosynostosis, selected by clinical or molecular geneticists as being high-priority cases, and in whom prior clinically driven genetic testing had been negative.

**Results:**

We identified likely associated mutations in 15 patients (37.5%), involving 14 different genes. All genes were mutated in single families, except for *IL11RA* (two families). We classified the other positive diagnoses as follows: commonly mutated craniosynostosis genes with atypical presentation (*EFNB1*, *TWIST1*); other core craniosynostosis genes (*CDC45*, *MSX2, ZIC1*); genes for which mutations are only rarely associated with craniosynostosis (*FBN1*, *HUWE1*, *KRAS*, *STAT3*); and known disease genes for which a causal relationship with craniosynostosis is currently unknown (*AHDC1*, *NTRK2*). In two further families, likely novel disease genes are currently undergoing functional validation. In 5 of the 15 positive cases, the (previously unanticipated) molecular diagnosis had immediate, actionable consequences for either genetic or medical management (mutations in *EFNB1*, *FBN1*, *KRAS*, *NTRK2, STAT3*).

**Conclusions:**

This substantial genetic heterogeneity, and the multiple actionable mutations identified, emphasises the benefits of exome/whole genome sequencing to identify causal mutations in craniosynostosis cases for which routine clinical testing has yielded negative results.

## Introduction

Accurate molecular classification is critical for the clinical management, counselling and prognosis of individuals with suspected monogenic diseases, particularly where early diagnosis and intervention would substantially influence decision-making by the clinician or family. Traditional phenotypically guided genetic testing may fail to identify rarer causes of disease as many conditions have a highly variable clinical presentation. The use of next-generation sequencing (NGS) technologies to interrogate the exome sequence (ES) or whole genome sequence (WGS) may circumvent some of these difficulties since these approaches are agnostic to the underlying genetic cause.^1–3^

Craniosynostosis, a condition that affects ∼1 in 2250 births,^4^ ^5^ is defined as the premature fusion of one or more of the normally patent cranial sutures, a consequence of disruption in the coordinated patterning, proliferation and differentiation of these tissues.^6^ It has a highly heterogeneous and complex aetiology with contributions from monogenic, chromosomal, polygenic and environmental factors all playing a role.^7^ ^8^ Craniosynostosis most commonly occurs in isolation, but a minority of cases are associated with additional clinical features as part of a syndrome, probably reflecting the co-option of pleiotropic signalling pathways to pattern and maintain the suture; association with >100 human syndromes has been reported.^9^ An underlying genetic cause can be identified in ∼24% of cases, with mutations in just six genes (in decreasing order of frequency: *FGFR2*, *FGFR3*, *TWIST1*, *TCF12*, *ERF* and *EFNB1*) together accounting for over three-quarters of monogenic diagnoses.^10–19^ At least 52 other genes have been identified as recurrently mutated in craniosynostosis,^8^ ^20^ but these rarer targets do not tend to be included in molecular testing panels unless indicated by specific clinical features. Craniosynostosis may also present as a low-frequency association with intellectual disability syndromes, possibly related to disturbed maintenance of suture patency.^8^

To evaluate the utility of ES and WGS in this context, we used these technologies to search for a molecular diagnosis in 40 patients with craniosynostosis who had previously been evaluated using existing routine molecular testing, without a diagnosis being made. Cases were identified as being of high priority for further investigation either by craniofacial clinical geneticists or by laboratory scientists specialising in craniofacial molecular diagnostics. From this cohort, we identified an underlying molecular lesion in 15 (37.5%) families. We document several cases where the mutation identified either did not obviously fit with the original clinical diagnosis or where identification of the causal mutation would have proved difficult using traditional methods of molecular testing. We highlight five families for which diagnosis has immediately impacted on clinical management or counselling. This work illustrates the molecular diversity of causes of craniosynostosis and the added value that can be gained by a comprehensive diagnostic approach in difficult cases.

## Subjects and methods

### Ethics statement and prior clinical investigation

Written, informed consent for genetic research and publication of clinical photographs was obtained by the referring clinicians and their research teams. DNA was extracted from whole blood. Genetic testing by a clinically accredited laboratory was guided by the judgement of the referring clinician and diagnostic laboratory but usually included dideoxy sequencing of *TWIST1*, *TCF12* and *ERF* (entire gene), *FGFR2* and *FGFR3* (regions enriched for craniosynostosis-associated mutations) and multiplex-ligation-dependent probe amplification of *TWIST1* to exclude heterozygous deletions.^7^ ^17^ ^18^ Since chromosomal abnormalities account for 13–15% of genetic diagnoses in craniosynostosis,^10^ ^17^ a karyotype or array comparative genomic hybridisation was undertaken in the majority of cases.

### Cohort description

Subjects with craniosynostosis, who had previously been investigated by molecular genetic testing with normal findings, were identified either by four clinical geneticists with specialist expertise in craniosynostosis (n=36) or by clinical laboratory scientists working in a specialist genetic diagnostic service (n=20) for further investigation. Clinical geneticists were asked to prioritise cases thought most likely to have a genetic diagnosis (based on presence of syndromic features, multiple sutures affected, consanguinity or positive family history, and lack of any obvious environmental predisposition), particularly where there were active issues with genetic counselling. Twelve of the cases initially identified by clinical geneticists were excluded either because further targeted genetic testing identified a monogenic cause (one case each with mutations in *ERF*,^18^ *FGFR2*^11^ and *FLNA,*^21^ and two with *IL11RA* mutations),^22^ where enrolment into the Deciphering Developmental Disorders study 3 was considered more appropriate (n=5) or where samples were not available for analysis (n=2). The remaining cases (n=24), together with parental samples (where available), were enrolled into the craniofacial research study following informed consent. Sequencing comprised 12 singletons, 1 parent–child duo, 10 parent–child trios and 1 trio comprising three affected individuals. Similar criteria were used by clinical laboratory scientists to prioritise 20 cases for ES; however, clinical information tended to be more limited and availability of sufficient stored DNA sample was often a decisive factor for case inclusion. For the laboratory samples, consent for research investigation was sought secondarily to enable the entire exome to be interrogated; two cases were excluded because consent was not obtained. In 16 of the remaining 18 laboratory diagnostic cases, ES was performed on the proband only; in one instance, the exome of an affected sibling was already available, and in another the family trio subsequently had WGS. Two duplicate families (29 and 34) identified by both clinicians and laboratory scientists are listed under the latter category in table 1, which summarises the patterns of cranial suture involvement and syndromic features of the final total of 40 patients/families analysed, with further details in online supplementary table S1. In general, cases referred by clinical geneticists exhibited higher proportions of multisuture involvement and syndromic features (table 1). The significance of differences between two groups was calculated using Fisher's exact test.

10.1136/jmedgenet-2016-104215.supp1 supplementary table 1

**Table 1:** Cranial suture involvement in patients recruited for exome sequence/whole genome sequence

	Non-syndromic		Syndromic		Combined	
	Total	Mutation positive	Total	Mutation positive	Total	Mutation positive
Clinical genetic cases						
Metopic	0	0	2	2	2	2
Sagittal	0	0	0	0	0	0
Unicoronal	0	0	1	0	1	0
Bicoronal	2	0	2	2	4	2
Multisuture	3	1	12	5*	15	6
Total	5	1	17	9	22	10
Molecular genetic cases						
Metopic	0	0	0	0	0	0
Sagittal	0	0	2	0	2	0
Unicoronal	5	1	1	1	6	2
Bicoronal	2	0	2	1*	4	1
Multisuture	1	0	5	2	6	2
Total	8	1	10	4	18	5

*Includes likely novel disease gene, still undergoing validation.

### Exome and whole genome sequencing

Exome capture of DNA from patients was carried out using the TruSeq v2 (Illumina), SureSelect Human All Exon Kit v4/v5 (Agilent) or SeqCap EZ Human Exome Library v2.0 (NimbleGen) following the manufacturer's instructions. We generated a library for each sample using DNA extracted from whole blood; usually we employed 3 μg DNA, except for SureSelect v5 processed samples from families 8, 16 and 17 only, for which we used 200 ng DNA. ES was performed on an Illumina HiSeq 2000 or 4000, with 75  or 100 bp paired-end reads. WGS was performed on 5 μg DNA extracted from blood by Complete Genomics (a BGI Company) and analysed as previously described.^23^

### Bioinformatic analysis

ES reads were mapped to the GRCh38 reference genome with Bowtie 2^24^ and removal of artefacts (unmapped sequences, duplicate PCR products and likely pseudogene sequences) using custom Perl scripts.^18^ Variants were called using SAMtools v1.1^25^ and Platypus v0.5.2.^26^ Sequence reads from WGS were mapped to the GRCh37 reference genome and analysed as previously described.^23^ The pathogenicity of each variant was given a custom deleterious score based on a six-point scale,^27^ calculated using output from ANNOVAR.^28^ Variants predicted to affect splicing were assigned a deleterious score based on MaxEntScan score differences,^29^ and the relationship of variants present in known disease-causing genes analysed for pathogenicity using ClinVar.^30^ Variants with minor allele frequency (MAF) >1% in dbSNP or ExAC were removed, and remaining variants examined manually by visualisation in GBrowse (hg37/hg38).^31^

In families comprising parent–child trios with a sporadic affected individual, we evaluated variants based on all likely modes of inheritance; de novo mutation, recessive (homozygous and compound heterozygous) and X-linked hemizygous variants (in males). For cases in which the parents were known to be related, the proband was usually sequenced as a singleton and the data interrogated for homozygous changes. For all samples, data were analysed for variants in 57 genes recurrently mutated in craniosynostosis^8^ and 1313 genes curated as being mutated in developmental disorders.^3^

### Variant validation

Confirmation of variants was carried out by dideoxy sequencing or restriction digest of genomic PCR amplification products. Primer sequences and conditions are detailed in online supplementary table S2. Amplification products were sequenced using the BigDye Terminator v3.1 cycle sequencer system (Applied Biosystems) and visualised using BioEdit Sequence Alignment Editor (Ibis Biosciences) and Mutation Surveyor Software (SoftGenetics).

10.1136/jmedgenet-2016-104215.supp2 supplementary table 2

Where a previously undescribed de novo variant was identified in a singleton sample (ie, *HUWE1*), correct sample relationships of the trio were checked by demonstrating consistent inheritance of nine microsatellite loci (*D1S2826*, *D3S1311*, *D5S2027*, *D6S1610*, *D9S158*, *D10S548*, *D13S1265*, *D14S280* and *D18S474*), labelled with 6-FAM fluorescent tags.

## Results

### Overview of molecular findings

We used either ES (n=37) or WGS (n=3) to seek a causative mutation in the two patient cohorts. In the cases from clinical geneticists (n=22), interpretation was often assisted by sequencing samples from unaffected parents or affected first-degree relatives; 23 additional samples were sequenced in this group (see online supplementary table S1). For most cases from the diagnostic laboratory, samples from relatives were either unavailable or not consented for analysis of ES/WGS (as described above) and, with two exceptions (total of three additional samples sequenced), these cases were analysed as singletons.

Mutations considered to be clinically significant (for the diagnosis of craniosynostosis and/or another genetic disorder) were identified in 15 of the 40 patients (37.5%), including two cases associated with putative novel disease genes that are still undergoing validation (table 2). Significantly more positive diagnoses were found in syndromic (13/27) than non-syndromic (2/13) patients (one-tailed p=0.046). The number of mutations identified in patients recruited via clinical geneticists (including the two duplicate ascertainments) was higher (11/24; 46%) compared with those recruited via the diagnostic laboratory (5/18; 28%), likely a consequence of more rigorous clinical selection and inclusion of sequencing data from a greater number of additional family members in these cases; however, the difference was not significant (one-tailed p=0.19). A positive diagnosis was obtained in a higher proportion of families in which multiple individuals were sequenced (6/14; 43%) than when singletons were sequenced (9/26; 35%), but this difference was also not significant. When the case solution rate was analysed in terms of total samples sequenced, sequencing of multiple individuals in a family appeared less cost-effective (six solved using a total of 40 ES/WGS; an efficiency of 0.43 per exome/genome sequenced compared with singleton sequencing).

**Table 2:** Summary details of patients with a positive genetic diagnosis

Family	Sex	GENE	Mutation		Inheritance	Ref	CRS	Clinical features†
3	F	*CDC45*	c.[226A>C];[469C>T]	p.[N76H];[R157C]	Compound heterozygous	32	BC	Short stature, thin eyebrows, anteriorly placed anus
4	M	*IL11RA*	c.[886C>T];[886C>T]	p.[R296W];[R296W]	Homozygous	22	P	Exorbitism, intellectual disability, atopy. ?Crouzon syndrome
7	F	Novel‡			Homozygous		P	Mid-face hypoplasia, corneal ulceration, scoliosis, severe respiratory tract infections/bronchiectasis, mild–moderate developmental delay
9	M	*IL11RA*	c.[98dupC];[98dupC]	p.[G34fs*39];[G34fs*39]	Homozygous	–	S, BC	Crouzonoid facies, mild developmental delay, dental anomalies, patent ductus arteriosus, atrial septal defect, umbilical hernia
1o	M	*MSX2*	c.443C>T	p.P148L	Heterozygous (from affected mother)	33 34	BC	Mild learning difficulties, short, broad thumbs, 5th finger clinodactyly, thick hair, squint and hydrocoele
11	M	*FBN1*	c.8226+5G>A	Splice	De novo	35	S, M	Exorbitism, ligamentous laxity, recurrent inguinal herniae, tall stature; lens subluxation and mild aortic dilatation aged 8 years
14	M	*HUWE1*	c.328C>T	p.R110W	De novo	2	M	Facial dysmorphism, dental anomalies, pectus excavatum, scoliosis, long palms, Chiari malformation, moderate–severe intellectual disability
16	M	*ZIC1*	c.1101C>A	p.C367*	Suspected de novo§	–	S, BL	Microcephaly, asymmetric ventriculomegaly, possible abnormalities on MRI brain imaging
18	M	*TWIST1*	c.350A>T	p.E117V	De novo	–	M	Hypertelorism, wide anterior fontanelle, upper eyelid colobomas, pseudoproptosis, dysplastic cupped ears, syndactyly of fingers, bilateral talipes, bilateral undescended testes, imperforate anus, hypertrichosis
21	F	*KRAS*	c.40G>A	p.V14I	De novo	36	P	Exorbitism, cloverleaf skull
23	F+M	Novel¶			Compound heterozygous		BC	Bilateral superior vena cava, dilated cardiomyopathy, rudimentary right thumb, duplex kidney, anterior anus, bilateral inguinal herniae, growth deficiency
24	F	*AHDC1*	c.2373_2374delTG	p.C791fs*57	De novo	37	BC, M	Moderate developmental delay, hoarse cry
25	F	*EFNB1*	c.325C>T	p.R109C	Paternal	38	RC	Hypertelorism
29	M	*STAT3*	c.1915C>T	p.P639S	De novo	39	P	Crouzonoid appearance, mild global developmental delay; necrotising pneumonia and bronchopleural fistula aged 3 years
37	F	*NTRK2*	c.1330G>T	p.G444*	Suspected de novo§	–	LC	Facial asymmetry, progressive onset of aggressive outbursts, ritualised behaviours and language delay, hyperphagic obesity, streak ovaries

†See online supplementary table S1 for detailed information.

‡Gene identity confirmed by functional testing (manuscript submitted).

§Father's sample not available for analysis.

¶Gene identity supported by similar case found on GeneMatcher; functional testing ongoing.

CRS, sutures fused in craniosynostosis: BC, bicoronal; BL, bilambdoid; LC, left coronal; LL, left lambdoid; M, metopic; P, pansynostosis; RC, right coronal; RL, right lambdoid; S, sagittal.

Table 2 summarises the 13 cases or families with mutation in a validated disease gene. Seven of the mutations identified (in *AHDC1*, *EFNB1*, *FBN1*, *IL11RA*, *KRAS*, *MSX2*, *STAT3*) were previously reported; in two instances (*CDC45* and *HUWE1*), the patients contributed to the first reported disease gene identification;^2^ ^32^ and in four cases, the mutations are newly identified and help to extend the genotype–phenotype spectrum (mutations in *IL11RA*, *NTRK2*, *TWIST1*, *ZIC1*). The associated phenotypes are summarised in table 2, and complete details are provided in online supplementary table S1. Aside from the importance of a molecular diagnosis to end the diagnostic odyssey and to enable precise genetic counselling (with appropriate estimation of recurrence risk and testing of at-risk family members), in five families the diagnosis had unexpected, actionable consequences for immediate clinical management. Details of these latter cases are provided as brief case reports to illustrate the range of diagnostic and management issues encountered; more complete descriptions are provided as online supplementary case reports.

10.1136/jmedgenet-2016-104215.supp3 supplementary case reports

### Case reports

#### Family 11: *FBN1* mutation

**Figure 1:**
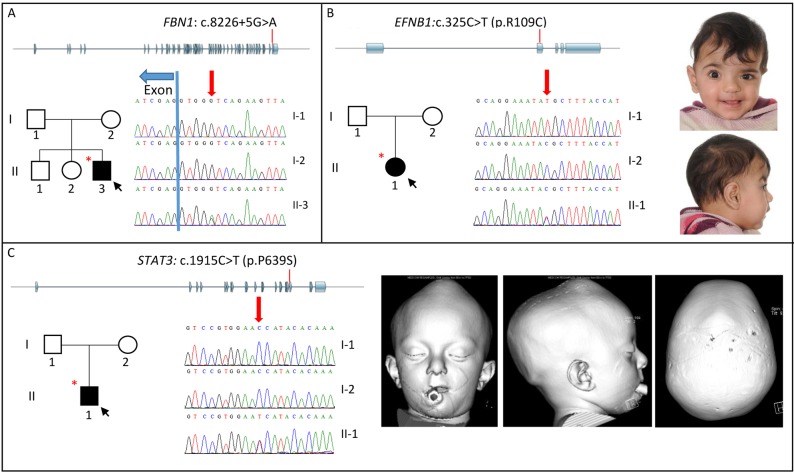
Family pedigrees, clinical photographs/3D-CT scans and sequencing traces of families with mutations identified in *FBN1* (A), *EFNB1* (B) and *STAT3* (C). Each panel shows the location of the mutation (red line) within the gene structure (exons in blue), family pedigree (affected individuals are in black, black arrow depicts the proband and individuals selected for exome sequence/whole genome sequence are indicated with a red asterisk), sequence traces of indicated individuals (red arrow indicates position of mutation) and clinical photographs (B) and 3D-CT scans (C) of affected individuals. Note facial asymmetry associated with right unicoronal synostosis in patient with *EFNB1* mutation (B); there is moderate hypertelorism, but the grooving of the nasal tip usually observed in craniofrontonasal syndrome is absent. In the patient with the *STAT3* mutation (C), images with soft tissue windows (left and centre) show exorbitism, mid-face hypoplasia and vertex bulge; image with bone windows (right) shows fusion of all sutures of the skull vault.

This boy (II-3 in figure 1A) was initially diagnosed with Shprintzen-Goldberg syndrome based on the combination of sagittal synostosis, blue sclerae, micrognathia, ligamentous laxity, bilateral recurrent inguinal herniae, tall stature and mildly abnormal aortic contour on echocardiography. However, sequencing of *SKI*, in addition to *TGFBR1* and *TGFBR2*, did not reveal any mutations.^40^

ES was performed on the proband only. Concomitantly, the referring clinician reported that the patient, now aged 8 years, had presented with subluxed lenses; together with the aortic findings, this suggested possible Marfan syndrome (MFS). Scrutiny of the ES data revealed two rare heterozygous variants in the *FBN1* (Fibrillin 1) gene, c.2615A>G (p.Lys872Arg) and c.8226+5G>A. Dideoxy-sequencing of parental samples showed that whereas the p.Lys872Arg substitution had been inherited from the unaffected father (not shown), the c.8226+5G>A variant had arisen de novo (figure 1A). The c.8226+5G>A variant, which was reconfirmed in a diagnostic laboratory, has been identified previously in a patient with a progeroid variant of MFS;^35^ a different mutation of the same splice site (c.8226+1G>A) was shown to cause skipping of the upstream exon, introducing a frameshift and premature stop codon.^41^

Confirmation of the molecular diagnosis of MFS has triggered a programme of lifelong monitoring owing to the association with progressive aortic dilatation; aged 10 years, mild aortic root dilatation was observed (Z score=3.02). Of note, craniosynostosis is an extremely rare but previously recognised association of MFS.^42^ ^43^

#### Family 21: *KRAS* mutation

This girl presented neonatally with a cloverleaf skull appearance. 3D-CT revealed synostosis of multiple cranial sutures. There were no additional syndromic features and cardiac examination was normal. Raised intracranial pressure (ICP) was documented, requiring a posterior vault expansion with springs, performed at the age of 3 months, and insertion of a right parietooccipital ventriculoperitoneal shunt.

WGS of the parent–child trio identified a heterozygous de novo mutation (c.40G>A encoding p.V14I) in *KRAS*, which has been reported previously in patients with Noonan syndrome.^36^ In the light of the exome results, an echocardiogram was performed, which was normal at almost 3 years of age, but she will continue cardiac surveillance. A coagulation screen has been normal but recommendations have been made to repeat it prior to any future surgery. Craniosynostosis is a rare but previously recognised complication of Noonan syndrome, being particularly associated with *KRAS* mutations.^44^

#### Family 25: *EFNB1* mutation

The female proband (II-1 in figure 1B), the first-born child to healthy parents with no relevant family history, was noted to have facial asymmetry at birth. Physical examination showed hypertelorism and ridging over the right coronal suture. A 3D-CT scan confirmed right coronal synostosis. She underwent a fronto-orbital advancement and remodelling (FOAR) procedure aged 15 months.

ES identified a heterozygous mutation (c.325C>T; p.Arg109Cys) in the X-linked *EFNB1* (ephrin-B1) gene, previously reported in a patient with craniofrontonasal syndrome (CFNS).^38^ Dideoxy sequencing of the parents showed that the clinically unaffected father (I-1) was hemizygous for the same variant (figure 1B). CFNS presents a paradoxical pattern of severity for an X-linked disorder, with heterozygous females more severely affected than hemizygous males, who can be non-penetrant.^45^ The result predicts that 100% of female children of the father would be expected to exhibit CFNS and/or craniosynostosis, and the couple have elected to enrol in a programme of preimplantation genetic diagnosis (PIGD), selecting only male embryos for uterine transfer.

#### Family 29: *STAT3* mutation

This boy (II-1 in figure 1C) presented to the craniofacial unit at 2 years of age with mild mid-facial hypoplasia, a short nose with a convex ridge, exorbitism and mild global development delay; 3D-CT scan demonstrated fusion of all of the cranial sutures with convexity of the closed anterior fontanelle, prominent ventricles and crowded basal cisterns. At the age of 2 years 2 months, ophthalmological assessment showed bilateral papilloedema and invasive monitoring demonstrated significantly raised ICP. He underwent a posterior vault expansion with insertion of springs at the age of 28 months.

ES of the proband was performed. This identified a heterozygous c.1915C>T (p.Pro639Ser) mutation in the SH2 domain encoded by *STAT3* (signal transducer and activator of transcription 3), which was previously reported in a case of hyper-IgE/Jobs syndrome.^40^ Dideoxy-sequencing of the parents showed that the mutation had arisen de novo (figure 1C).

Upon feedback of this finding, it transpired that the proband had more recently presented at the age of 3 years 3 months with an upper respiratory tract infection, progressing to severe necrotising pneumonia with a pulmonary abscess and pneumatocoele. He developed a pneumothorax and bronchopleural fistula; following two unsuccessful attempts at surgical resection, he required a right lower lobe segmentectomy. A large secundum atrial septal defect required patch closure at 5 years of age. Further immunological assessment demonstrated a markedly elevated total IgE of 3091 kU/L (normal range 0–52). He commenced prophylactic azithromycin and itraconazole and is awaiting a suitably matched donor for stem cell transplantation. Bone mineral density assessment was normal, but he takes multivitamin supplements including vitamin D and is under enhanced dental and skeletal surveillance.

#### Family 37: *NTRK2* mutation

The female proband presented with an asymmetric face at 12 months of age and left coronal synostosis was diagnosed on 3D-CT scan; there were no syndromic features. She underwent a FOAR procedure aged 17 months. On clinical follow-up at the age of 2 years 8 months, she was noted to have episodes of temper tantrums and was exhibiting speech and language delay. By the age of 6 years, she required a school statement indicating moderate learning difficulties. ES identified a heterozygous nonsense mutation (c.1330G>T; p.Gly444*) in *NTRK2*, encoding neurotrophic tyrosine receptor kinase, type 2, with a predicted loss of the entire intracellular tyrosine kinase domain. The proband's mother did not carry the mutation and the father was not available for analysis. Dominant mutations of *NTRK2* have been described in association with hyperphagic obesity associated with developmental delay (OBHD), and functional studies have pointed to haploinsufficiency as the likely pathogenic mechanism of disease associated with a previously identified p.Tyr722Cys substitution.^46^ ^47^

The discovery of the *NTRK2* mutation prompted a further endocrinology assessment at the age of 7 years 6 months. Her height was 135.2 cm (+2.04 SD), weight 46.2 kg (+3.19 SD) and body mass index 25.3 kg/m^2^ (+3.1 SD), consistent with a diagnosis of OBHD. She was noted to have a long-standing history of hyperphagia. The oral glucose tolerance test was normal; streak ovaries and uterus were evident on ultrasound scan. Management implications have included referral to a clinical psychologist and dietitian to address her eating behaviours, and regular monitoring for secondary complications including cardiovascular disease and diabetes.

## Discussion

We present, to our knowledge, the first investigation of the added value provided by ES or WGS in molecular genetic diagnosis of craniosynostosis, applied to two cohorts of patients (total of 40) identified as high-priority cases by clinical or laboratory geneticists following negative results from routine molecular genetic testing. We identified 13 mutations in 12 confirmed disease genes that we considered to be pathogenic. Seven of the particular DNA sequence changes found were previously reported, whereas an additional six are currently unique to the patients described here. The newly identified variants were considered pathogenic using evidence from population MAF data, predictive computational data and studies of aberrant function (either performed ourselves or published in the literature), according to guidelines from the Association for Clinical Genetic Science.^48^ In addition, we identified at least two likely novel disease-associated genes; further studies to corroborate these findings are ongoing, so details are not presented here. Including these latter cases, our overall success rate in identifying pathogenic mutations was 15/40 (37.5%), which is towards the upper end of the range usually quoted in ES/WGS analysis of other diseases with a major genetic component.^1–3^ We used a mixed strategy of sequencing both singletons and multiple family members; the latter strategy was associated with a higher success rate per family, but the former with a 2.3-fold higher success rate per sample sequenced. Although trio sequencing is the favoured design in many other ES/WGS studies because complete bioinformatic analysis of the data is more straightforward,^1–3^ singleton sequencing appears more cost-effective in a diagnostic setting.

Craniosynostosis comprises a very diverse group of disorders and its causes are correspondingly heterogeneous, with intrauterine fetal head constraint, reduced transduction of stretch forces from the growing brain owing to poor intrinsic growth, and polygenic background all likely to play substantial roles, in addition to monogenic causes.^8^ Since a major motivation of this work was to use NGS to identify novel disease genes in craniosynostosis, we selected cases suspected to have a genetic cause, based on positive family history, presence of additional syndromic features or multiple suture fusions, and for which clinically guided genetic testing had been normal. Although this strategy was successful, with at least four newly recognised disease genes for craniosynostosis being identified as part of this study (*CDC4*5^32^ and *HUWE1*,^2^ and two awaiting further corroboration), the major finding presented here is that NGS is very valuable for diagnosis of a long ‘tail’ of rare genetic associations with craniosynostosis. All of these positive diagnoses have made a critical difference to genetic counselling, with some having broader management implications.

**Figure 2:**
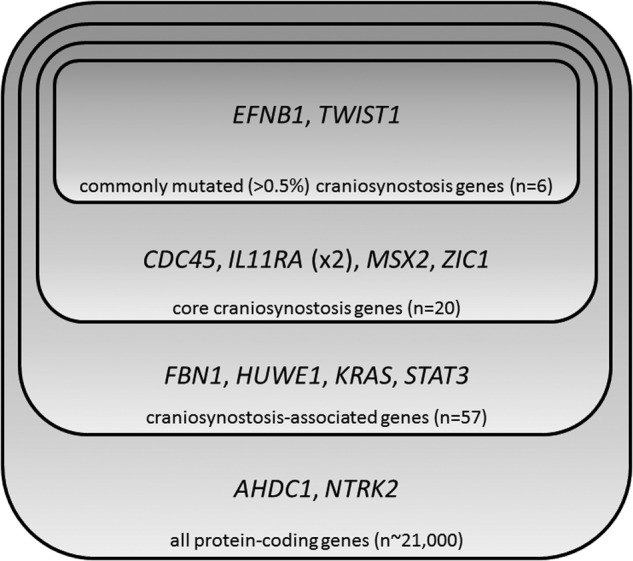
Identified mutations and their association within the classification of craniosynostosis-associated genes proposed by Twigg and Wilkie.^8^

To understand why a genetic diagnosis in craniosynostosis may elude standard molecular diagnostic testing, we categorised each additional diagnosis in terms of the molecular genetic framework recently presented by Twigg and Wilkie.^8^ These authors identified 57 genes as recurrently mutated in craniosynostosis, of which they categorised 20 as ‘core genes’ (craniosynostosis present in >50% of patients with specific categories of mutation in that gene), while mutations in the remaining 37 genes were associated with craniosynostosis in only a minority of cases. The core genes could be further subdivided into six with mutations each accounting for >0.5% of all craniosynostosis and 14 more rarely mutated genes. Figure 2 summarises how the 13 identified mutations are classified according to this framework. While, not surprisingly, the number of mutations identified, as a proportion of total genes in the category, rose progressively as the pathogenic hierarchy was ascended, there were multiple genes in each category. This includes two genes (*NTRK2, AHDC1*), for which we are unaware of any previous association with craniosynostosis; it is unclear whether the co-occurrence of the mutation and sentinel phenotype is causally linked (potentially through the adverse effect of the mutation on brain development)^8^ or simply coincidental.

In the cases found to have mutations in the ‘core’ genes, the question arises why these were not identified by testing within the routine diagnostic service. In the patients with *EFNB1* and *TWIST1* mutations, the diagnosis was missed because analysis of the relevant gene had not been requested by the clinician; either because of an unusually mild presentation (*EFNB1*, figure 1B) or a severe, atypical presentation (*TWIST1*). Of the other core genes, homozygous mutations in *IL11RA*, first described in 2011,^22^ are increasingly recognised, especially in consanguineous families from the Indian subcontinent, and clinical diagnostic testing has now been introduced in the UK (http://ukgtn.nhs.uk/uploads/tx_ukgtn/CRSDA_IL11RA_GD_Sept_14.pdf). Although a specific heterozygous mutation of *MSX2* encoding Pro148His was the first molecular lesion to be described in craniosynostosis in 1993,^49^ only two further families (both segregating p.Pro148Leu) have subsequently been reported worldwide,^33^ ^34^ and the family described here (also with p.Pro148Leu) is the first known in the UK. Finally, the *CDC45* patient contributed to the recent identification of mutations in this gene,^32^ and the patient with the *ZIC1* mutation extends the currently described genotype–phenotype correlation,^50^ being the first with a mutation in exon 2 and also the first with involvement of the sagittal suture instead of the originally described presentation with bilateral coronal synostosis.

As in any branch of genetic medicine, achieving a precise molecular diagnosis has immediate implications for genetic counselling, both in terms of recurrence risk and for targeted preventive measures. The common craniosynostosis syndromes all show dominant patterns of inheritance, so that when parents are clinically unaffected, empiric recurrence risks are low (around 5%).^7^ ^51^ Our cohort illustrates two scenarios where providing the standard genetic advice would substantially underestimate recurrence risks. In family 25 (*EFNB1* mutation), the clinically unaffected father was shown to be hemizygous for the mutation originally identified in his daughter, indicating that the risk of CFNS in future female children is 100%. In family 3 (*CDC45* mutation), the autosomal-recessive inheritance of this disorder raises the recurrence risk for children of the unaffected parents to 25%. Both findings have affected reproductive decision-making; one family is seeking PIGD, whereas in the other, the option not to have further children is being considered.

In addition to the genetic implications, in several cases (mutations in the *FBN1*, *KRAS*, *NTRK2* and *STAT3* genes), the molecular diagnosis has had immediate implications for clinical management, as described in the case reports, so genetic diagnosis was additionally important. Mutations in each of these genes are only infrequently associated with craniosynostosis; indeed, the presence of the craniosynostosis may have delayed correct diagnosis by laying a confusing trail. As a result of the molecular diagnosis, appropriate, potentially life-saving monitoring has been instigated. The high apparent rate of rare actionable mutations identified in complex craniosynostosis without an obvious diagnosis may reflect developmental pleiotropy of signalling in the cranial sutures, with multiple pathways, co-opted from more ancient uses in embryogenesis, implicated at different stages of suture development.^8^

In summary, our findings illustrate the considerable added value provided by ES/WGS to the precise diagnosis of patients with craniosynostosis suspected to have a genetic cause, but where routine testing has failed to elucidate this. As technologies improve, a strategic question in molecular diagnostics is whether it is preferable to extend panel tests to more genes or whether to opt directly for ES/WGS. Aside from the most common disease-associated mutations, for which targeted testing currently remains cost-effective, we propose, based on the distribution of mutations identified (figure 2), together with the substantial burden of actionable findings (case reports), a low threshold for implementing ES/WGS. An additional benefit of this strategy is that it may identify new disease loci,^2^ ^32^ enabling improved diagnostic and management strategies in the future.

## Gene accession numbers

*AHDC1* (NM_001029882), *CDC45* (NM_003504), *EFNB1* (NM_004429), *FBN1* (NM_000138), *HUWE1* (NM_031407), *IL11RA* (NM_001142784), *KRAS* (NM_033360), *MSX2* (NM_002449), *NTRK2* (NM_001007097), *TWIST1* (NM_000474), *STAT3* (NM_139276), *ZIC1* (NM_003412).

## Web resources

MaxEntScan (http://genes.mit.edu/burgelab/maxent/Xmaxentscan_scoreseq.html)

NNSPLICE/BDGP (http://www.fruitfly.org/seq_tools/splice.html)

dbSNP (http://www.ncbi.nlm.nih.gov/SNP/)

ExAC (http://exac.broadinstitute.org/)

GeneMatcher (https://genematcher.org/)

ClinVAR (http://www.ncbi.nlm.nih.gov/clinvar/)

LOVD (http://databases.lovd.nl/shared/genes/ZIC1)
