# Supplementary material for: Diagnostic value of exome and whole genome sequencing in craniosynostosis
Source: J Med Genet. 2016 Nov 24;54(4):260–8. doi: 10.1136/jmedgenet-2016-104215 (PMC5366069; doi:10.1136/jmedgenet-2016-104215)
Supplement: supplementary table 1 [file jmedgenet-2016-104215supp001.pdf]

Table S1: Extended phenotypic information for samples processed for ES and WGS.

Family #	Sample	Sex	Sample ID	Referring Clinic[+]	Seq method	Library kit	Syndromic[$]	Suture involvement	Additional clinical manifestations	Family history	Population (if non-European)	Mutation Positive			
**Clinical genetic cases**												**Gene**	**Genomic**	**Protein**	**Inheritance/allelic state**
**1**	Proband	M	5490	BCH	Exome	TruSeq	Y	P	Learning disability, prominent eyes, hypertelorism, ?submucous cleft, mild syndactyly fingers, limited pronation both arms, broad great toes, perimembraneous venticular septal defect, coarctation of aorta, choanal atresia, bilateral inguinal hernia, hearing loss (bone anchored hearing aids).	Mother with similar facial features, atrial septal defect, mild learning disability, no craniosynostosis		-	-	-	-
**2**	Proband	F	5383	BCH	Exome	TruSeq	Y	S, BC	Exorbitism, abnormal posture R arm. long, slender fingers. Evolving pansynostosis and recurrent synostosis post surgery			-	-	-	-
	Unaffected mother	F	5406												
	Unaffected father	M	5407												
**3**	Proband	F	4964	GOS	Exome	TruSeq	Y	BC	Short stature, thin eyebrows, anteriorly placed anus			*CDC45*	c.[226A>C];[469C>T]	p.[N76H];[R157C]	Compound heterozygous
**4**	Proband	M	5627	GOS	Exome	TruSeq	Y	P	Exorbitism, intellectual disability, atopy (eczema, asthma, dietary allegies). ?Crouzon syndrome	Parents 1st cousins	South Asian	*IL11RA*	c.[886C>T];[886C>T]	p.[R296W];[R296W]	Homozygous
**5**	Proband	M	5630	GOS	Exome	TruSeq	Y	S, BC	Exorbitism, gum hypertrophy, disrupted dental eruption. Hairy external auditory meatus			-	-	-	-
	Unaffected mother	F	5631												
	Unaffected father	M	5632												
**6**	Proband	F	5626	GOS	Exome	TruSeq	Y	S, BL	Upslanting palpebral fissures, mild exorbitism, hypertelorism, mild ptosis, mild 5th finger clinodactyly, anterior			-	-	-	-

									anus, dilated cardiomyopathy						
**7**	Proband	F	5169	Liv	Exome	TruSeq	Y	P	Mid-face hypoplasia, obstructive sleep apnoea, corneal ulceration, progressive scoliosis, multiple severe respiratory tract infections with bronchiectasis, mild-moderate cognitive impairment	Parents 1st cousins	South Asian	#	-	-	-
**8**	Proband	F	5333	BCH	Exome	TruSeq	Y	S, LC, LL	None	Affected father and sister also analysed		-	-	-	-
	Affected father	M	5335			ss v5	Y	S	Headshape compatible with sagittal synostosis, no imaging confirmation						
	Affected sibling	F	6931				Y	BC	Ollier disease, scoliosis, non-progressive muscular dystrophy (myopathic pseudo dystrophic changes on muscle biopsy)						
**9**	Proband	M	5520	BCH	Exome	ss v5	Y	S, BC	Crouzonoid facies, mild developmental delay, delayed dental eruption, small pointed teeth, class III malocclusion, patent ductus arteriosus, atrial septal defect, umbilical hernia	Parents 1st cousins	South Asian	*IL11RA*	c.[98dupC];[98dupC]	p.[G34fs*];[G34fs*]	Homozygous
	Unaffected mother	F	5518									*IL11RA*	c.98dupC	p.G34fs*	Heterozygous
	Unaffected father	M	5519									*IL11RA*	c.98dupC	p.G34fs*	Heterozygous
**10**	Proband	M	6306	BCH	Exome	ss v5	Y	BC	Mild learning difficulties, slightly short, broad thumbs, 5th finger clinodactylly, thick hair. Squint and hydocele requiring surgery at 7yrs of age.	Affected mother also analysed		*MSX2*	c.443C>T	p.P148L	Heterozygous
	Affected mother	F	5326				Y	BC	Short 5th fingers, thick hair			*MSX2*	c.443C>T	p.P148L	Heterozygous
**11**	Proband	M	5219	GOS	Exome	ss v5	Y	S, M	Exorbitism, hypoplastic midface, downslanting palpebral fissures, blue sclerae, micrognathia, prominent nasal bridge, ligamentous			*FBN1*	c.8226+5G>A	(splice site)	De novo

									laxity, recurrent inguinal herniae, tall stature; lens subluxation and mild aortic dilatation aged 8 years						
**12**	Proband	M	6772	GOS	Exome	ss v5	Y	BC, M	Brachycephaly, slightly deviated septum, mild exorbitism, slightly deviated halluses		South Asian	-	-	-	-
	Unaffected mother	F	6379												
	Unaffected father	M	6380												
**13**	Proband	M	6796	GOS	Exome	ss v5	N	P	High anterior hairline, hypertelorism, divergent squint, mild exorbitism, left upper eyelid retention cyst, wide eyebrows			-	-	-	-
	Unaffected father	M	5891												
	Unaffected mother	F	5892												
**14**	Proband	M	6589	Liv	Exome	ss v5	Y	M	Flat mid-face, down-slanting palpebral fissures, low set ears, facial asymmetry, retracted pre-maxilla, right choanal stenosis, micrognathia, peg-like teeth with conical incisors, absent lateral incisors, pectus excavatum, scoliosis, long palms, slight digital shortening, mild 4/5 syndactyly of toes, Chiari malformation, moderate - severe learning difficulties.		Middle East	*HUWE1*	c.328C>T	p.R110W	De novo
**15**	Proband	M	6463	GOS	Exome	ss v5	N	BC	Beta-Thalassaemia	Parents 1st cousins	South Asian	-	-	-	-
**16**	Proband	M	6966	GOS	Exome	ss v5	Y	S, BL	Microcephaly, asymmetric ventriculomegaly, possible abnormalities on MRI brain scan involving the corpus callosum, posterior fossa and frontal and perisylvian cortex			*ZIC1*	c.1101C>A	p.C367*	Heterozygous; absent in mother, father not available
**17**	Proband	M	6223	Liv	Exome	ss v5	N	P	None			-	-	-	
	Unaffected mother	F	6803												
	Unaffected father	M	6804												

**18**	Proband	M	5856	Liv	Exome	ss v5	Y	M	Hypertelorism, wide anterior fontanelle, upper eyelid colobomas, deficient bony orbits with pseudoproptosis, small low-set dysplastic cupped ears, 2,3,4 syndactyly of fingers, bilateral talipes, bilateral undescended testes, imperforate anus, hypertrichosis, mild-moderate learning disability.			*TWIST1*	c.350A>T	p.E117V	De novo
	Unaffected father	M	5854												
	Unaffected mother	F	5855												
**19**	Proband	M	5629	GOS	Exome	ss v5	Y	LC	Cleft soft palate, severe micrognathia, hypoplastic tongue with ankyloglossia, double outlet right ventricle (Fallots type), pulmonary artery stenosis, gastro-oesophageal reflux, small right pelvic kidney, partial 3/4 cutaneous syndactyly left hand and foot			-	-	-	-
	Unaffected father	M	5673												
	Unaffected mother	F	5674												
**20**	Proband	F	5285	BCH	Exome	ss v5	N	BC	None		South Asian	-	-	-	-
**21**	Proband	F	6701	GOS	Whole Genome	Complete Genomics	Y	P	Exorbitism, clover leaf skull			*KRAS*	c.40G>A	p.V14I	De novo
	Unaffected mother	F	6254												
	Unaffected father	M	6256												
**22**	Proband	M	6246	BCH	Whole Genome	Complete Genomics	Y	S, BC	Mild facial dysmorphism, mild bilateral ptosis, prominent eyes, small nose, short fingers, bilateral palmer creases. Recurrent raised intracranial pressure requiring repeat surgery, seizures pre-op and 9 months of age. Father has minor digit anomalies.			-	-	-	-
	Unaffected mother	F	5301												
	Unaffected father	M	5302												

**Molecular genetic cases**															
**23**	Proband	F	5561	Ox	Exome	ss v4	Y	BC	Bilateral superior vena cava, dilated cardiomyopathy, rudimentary right thumb, left hip dislocation, duplex kidney, anterior anus, bilateral inguinal herniae, growth deficiency			#			
	Affected sibling	M	6181			NimbleGen SeqCap EZ Exome v2.0	Y	BC	Prenatal diagnosis of intrauterine growth retardation, abnormal ductus venosus, right talipes equinovarus, hypospadias, selective termination at 32 weeks' gestation, healthy twin born and developed normally			#	-	-	-
**24**	Proband	F	6222	Liv	Whole Genome	Complete Genomics	Y	BC, M	Moderate developmental delay, hoarse cry			*AHDC1*	c.2373_2374delTG	p.C791fs*	De novo
	Unaffected mother	M	6183												
	Unaffected father	F	6184												
**25**	Proband	F	5928	Ox	Exome	NimbleGen SeqCap EZ Exome v2.0	N	RC	Hypertelorism		South Asian	*EFNB1*	c.325C>T	p.R109C	Heterozygous; inherited from hemizygous father
**26**	Proband	M	6082	Ox	Exome	NimbleGen SeqCap EZ Exome v2.0	N	RC	Strabismus			-	-	-	-
**27**	Proband	F	6122	Ox	Exome	NimbleGen SeqCap EZ Exome v2.0	N	RC	None			-	-	-	-
**28**	Proband	F	6030	Ox	Exome	NimbleGen SeqCap EZ Exome v2.0	N	LC	None			-	-	-	-

**29***	Proband	M	5762	GOS	Exome	Nimble Gen SeqCap EZ Exome v2.0	Y	P	Crouzonoid appearance, mild global developmental delay; 3 years old, necrotising pneumonia and broncho-pleural fistula		South Asian	*STAT3*	c.1915C>T	p.P639S	De novo
**30**	Proband	M	5657	Ox	Exome	Nimble Gen SeqCap EZ Exome v2.0	Y	S	Mild facial dysmorphism, Chiari malformation, mild developmental delay, duplication of *SH2B1* on array CGH			-	-	-	-
**31**	Proband	F	5944	Ox	Exome	Nimble Gen SeqCap EZ Exome v2.0	Y	S, LC	Left hemifacial hypertrophy, high anterior hairline, frontal bossing, low set ears, single palmar creases, mild bilatateral cutaneous syndactyly, 4/5 clinobrachydactyly of fingers, small toes; hypotonic with moderate speech and cognitive delay.			-	-	-	-
**32**	Proband	F	6136	BCH	Exome	Nimble Gen SeqCap EZ Exome v2.0	N	BC	3,4,5 clinodactyly of toes (medially deviated), mild hypertelorism, mother clinically normal			-	-	-	-
**33**	Proband	M	5322	Ox	Exome	Nimble Gen SeqCap EZ Exome v2.0	N	S, BL	None		African	-	-	-	-
**34***	Proband	M	6569	Liv	Exome	Nimble Gen SeqCap EZ Exome v2.0	Y	P	Low anterior hairline, synophrys, small mouth. Visual loss secondary to raised intracranial pressure; shunted. Empty sella. Moderate developmental delay and learning disability, attention deficit disorder, behavioural problems.			-	-	-	-
**35**	Proband	F	6568	GOS	Exome	Nimble Gen SeqCap	N	BC	None			-	-	-	-

						EZ Exome v2.0									
**36**	Proband	M	4332	Ox	Exome	Nimble Gen SeqCap EZ Exome v2.0	Y	S, M	Trigonocephaly associated with hypertelorism, small ventricular septal defect, mild expressive language deficit			-	-	-	-
**37**	Proband	F	4473	Ox	Exome	Nimble Gen SeqCap EZ Exome v2.0	Y	LC	Facial asymmetry, progressive onset of aggressive outbursts, ritualised behaviours and language delay, hyperphagic obesity, streak ovaries			*NTRK2*	c.1330G>T	p.G444*	Heterozygous; absent in mother, father not available
**38**	Proband	F	6721	BCH	Exome	Nimble Gen SeqCap EZ Exome v2.0	Y	BC	Developmental delay, congenital hip dislocation (treated with Spica), positional talipes, congenital dislocation left knee resolved spontaneously, midface hypoplasia, downslanting palpebral fissures, bilateral sensorineural hearing loss, seizures. Both parents have learning disability and father has epilepsy			-	-	-	-
**39**	Proband	F	6722	Ox	Exome	Nimble Gen SeqCap EZ Exome v2.0	N	LC	None			-	-	-	-
**40**	Proband	M	6368	Ox	Exome	Nimble Gen SeqCap EZ Exome v2.0	Y	S	None	Male sibling and maternal uncle also reported to have sagittal synostosis		-	-	-	-

S; sagittal, LC; Left Coronal; RC; Right Coronal, BC; Bicoronal, M; Metopic, LL; Left Lambdoid, RL; Right Lambdoid, BL; Bilambdoid, P; Pansynostosis

*Also prioritised by clinical geneticist

[$]Based on additional clinical features or positive family history

[#]Likely novel disease gene, still undergoing validation

[+]BCH, Birmingham Children's Hospital; GOS, Great Ormond Street Hospital; Liv, Alder Hey Children's Hospital; Ox, John Radcliffe Hospital.

[¥]Parental DNA unavailable.
