# Supplementary material for: Diagnostic value of exome and whole genome sequencing in craniosynostosis
Source: J Med Genet. 2016 Nov 24;54(4):260–8. doi: 10.1136/jmedgenet-2016-104215 (PMC5366069; doi:10.1136/jmedgenet-2016-104215)
Supplement: supplementary table 2 [file jmedgenet-2016-104215supp002.pdf]

Table S2: Primer sequences and conditions used for confirmation of variants[A]

GENE	FOR PRIMER SEQUENCE	REV PRIMER SEQUENCE	PRODUCT SIZE (bp)	TM (°C)	DMSO	DIGEST
CDC45	TAGTGATGAAGGAAAAGGGGCCTCCTCG	CTGTTCCCAGTCCCACAGGGTAGTCAG	314	63	N	-
	ACCACGTATGGTGTAACTCTGGTGCCTCAC	CTTGGCCTGGCAGGCTTCAGGATGAC	436			-
IL11RA	*(4) GTAGATGGTGGCTGGGAAGGCC	GCTAAGACAGGGCTGTAGGCAAACAAG	369	60	N	AvaI @ 37°C
	*(9) CAGGCTTTAGCCTCCCATCTCAGG	GGGACATGAGGACTAGGCAGAGTC	557	61	N	-
MSX2	TTGCTAATCCGCTCCTCTCTCTG	AAACAAGCATCCATCATGGAG	496	58	N	-
FBN1	ACTCTTTTAGGCCCAAGACTAG	AGCTCAGAACCCCTAACCAC	812	58	N	SmlI @ 55°C
	CACCAGAACTCTTGTACCACC	CAGAGGAAATGAGCAAGTATGTG	483	58	N	-
HUWE1	GATGCTGGACAGACAGTGGA	ACAGGGTACACAGGCTTTAACC	183	60	N	HpaII @ 37°C
ZIC1	TTTTAAGCTTGCAAAGTGCTAATCCTG	CCAAGAGAGCTCCTGCCTCAAAG	344	65	N	-
TWIST1	CGGCAAGAAGTCTGCGGGCTG	CCTGGCCGGCCTGCCGTCTGCCACCTGAGAG	490	63	Y	HaeII @ 37°C
KRAS[+]	GACTGAATATAAACTTGTGGTAGTTGG	TTGGATCATATTCGTCCACAA	101	55	N	-
AHDC1	CCGGAAGAATGGGACTCTG	CCGGTGAAGAGGTTTTGGC	282	58	N	-
EFNB1	(A) TTGTCCGCTTCCCTGGTTCT	TGCACCACTTAGAAGCTCCCACT	443	63	N	-
	(B) CCCCCAACCCTGAGGCTGACCATC	GAGTTAAGCCCAGGGAGAGAGCCAGAGG	367			
STAT3	ACAGGGTGTTCAGGGTCTC	CCTCCTTGGGAATGTCAGG	286	63	N	-
NTRK2	AGATGGATGGAGAGAGAGCTG	TGGAAAAGTGGGTAGTGCTG	598	64	N	-

[A]PCR amplifications were was performed in a 20 µl reaction containing 20 ng DNA, 15-50 mM Tris-HCl, 10-50 mM KCl, 0-55 mM $(NH_4)_2SO_4$, 2-2.5 mM $MgCl_2$, 100 µM each dNTP, 0.4-0.5 µM primers and 0.75 units of FastStart[TM] Taq DNA polymerase (Roche) with the addition of 10% DMSO (Sigma), where indicated. Cycling conditions consisted of an 8 min denaturation step at 95°C, followed by 35 cycles of 95°C for 30 s, annealing at 58-63°C for 30-60 s and extension at 72 °C for 30 s-1 min, with a final extension at 72 °C for 10 min. PCR reactions performed by the clinical lab for confirmation of variants (i.e *EFNB1*) were amplified in a 20 µl reaction using KAPA2G Fast HS readymix (KAPA Biosystems) with 10 mM $MgCl_2$, 10 µM each primer and 150-200 ng DNA. Cycling conditions consisted of a 3 min denaturation step at 95°C, followed by 35 cycles of 95°C for 10 s, 63°C for 10 s and 72°C for 1 s, with a final extension at 72°C for 10 min. Restriction digests were carried out in a total volume of 20 µl, containing 5 µl PCR product, 10 U of enzyme (NEB), 1x buffer solution and 1x bovine serum albumin (BSA) if necessary. Incubation was carried out at the specified temperature for 1 h-overnight.

[+]Mutation was confirmed diagnostically using pyrosequencing technology, on a PyroMark Q96 system with biotinylation of the reverse primer and the following pyrosequencing primer: 5'-CTTGTGGTAGTTGGAG-3'.

*Family ID, refer to Table S1
