# Supplementary material for: Diagnostic value of exome and whole genome sequencing in craniosynostosis
Source: J Med Genet. 2016 Nov 24;54(4):260–8. doi: 10.1136/jmedgenet-2016-104215 (PMC5366069; doi:10.1136/jmedgenet-2016-104215)
Supplement: supplementary case reports [file jmedgenet-2016-104215supp003.pdf]

Case Reports

Family 11: *FBN1* mutation. This boy (II-3 in Fig. 1A) is the third child of healthy parents from the Middle East, with no prior family history of craniosynostosis. He was born at 37 weeks' gestation (birth weight 2830 g (-0.25 SD); length 52 cm (+1.76 SD); OFC 33.4 cm (-0.03 SD)) and needed neonatal care for treatment of amniotic fluid aspiration. A clinical genetics opinion was requested because of dysmorphic features, a large fontanelle, and stridor caused by left vocal cord palsy. A skeletal survey and ophthalmology assessment were normal, and no unifying diagnosis was made.

On further evaluation at the age of 10 months, he was noted to have a long narrow face, scaphocephaly, large (3 cm x 4 cm) anterior fontanelle, blue sclerae, vertical dimple on the chin and hypermobility of his small joints. His length was 75 cm (75th centile), and developmental milestones were normal. A skeletal survey was repeated, and craniofacial investigation confirmed sagittal synostosis. A total calvarial remodelling procedure was performed at the age of 3 years 10 months. A diagnosis of Shprintzen-Goldberg syndrome (SGS; OMIM 182212) was proposed, based on the combination of sagittal synostosis, exorbitism, downslanting palpebral fissures, blue sclerae, prominent nasal bridge, midface hypoplasia, micrognathia, syndactyly of the fingers, ligamentous laxity, recurrent inguinal herniae and tall stature. Targeted sequencing of the *SKI* gene, [1] in addition to *TGFBR1* and *TGFBR2*, did not reveal any mutations. Echocardiography, when initially performed at 2 years 9 months, was normal; on repeat examination at the age of 4 years, requested because of the phenotypic overlap with Marfan syndrome (MFS; OMIM 154700), the cardiologist considered that the shape of the aorta was mildly abnormal (although the dimensions were within normal limits), and he continued to be followed up.

We performed WES of the proband as a singleton; concomitantly, the referring clinician reported that the patient, by now aged 8 years, had recently presented with subluxed lenses, a mild scoliosis and a mildly dilated aorta. His modified Ghent score was 7/20, suggesting a possible diagnosis of Marfan syndrome. Scrutiny of the WES data revealed two rare heterozygous variants in the *FBN1* (Fibrillin 1) gene, c.2615A>G encoding p.Lys872Arg and the splice site variant

c.8226+5G>A; Figure 1A). Dideoxy-sequencing of parental samples showed that whereas the p.Lys872Arg variant had been inherited from the unaffected father (not shown), the c.8226+5G>A had arisen *de novo* (Figure 1A). The c.8226+5G>A variant, which was re- confirmed in a diagnostic laboratory, has been identified previously in a patient with a progeroid variant of MFS; [2] a different mutation of the same splice site (c.8226+1G>A caused skipping of the associated exon, introducing a frameshift and premature stop codon. [3]

Confirmation of the molecular diagnosis of MFS has triggered a program of lifelong monitoring owing to the association with progressive aortic dilatation; aged 10 years, he has mild aortic root dilatation (Z score = 3.02). Of note, craniosynostosis is an extremely rare, but previously recognised association of MFS. [4, 5]

Family 21: *KRAS* mutation. This girl, born to healthy unrelated parents, presented neonatally with a cloverleaf skull appearance. Computed tomography (CT) scanning revealed synostosis of both coronal sutures, the posterior part of the sagittal, both lambdoids and partial sphenoidal synostosis. There were no additional syndromic features and cardiac examination was normal. Due to CT and ophthalmological evidence of raised intracranial pressure (ICP), a posterior vault expansion with springs was performed at 3 months of age; the springs were removed 3 weeks later owing to infection. Due to persisting raised ICP, a right parieto-occipital ventriculo-peritoneal shunt was inserted aged 7 months which has required a number of revisions. She was suspected to have Crouzon syndrome but targeted sequencing of *FGFR2*, *FGFR3*, *TWIST1*, *TCF12* and *ERF* was normal, as was *TWIST1* multiplex ligation-dependent probe amplification (MLPA) and array-CGH chromosome testing. Whole genome sequencing of the parent-child trio identified a heterozygous *de novo* mutation (c.40G>A encoding p.V14I) in *KRAS*. This mutation has been reported previously in patients with Noonan syndrome. [6] Although *KRAS* mutations only account for ~7% of the Noonan syndrome spectrum, [7] craniosynostosis has been reported in 6 patients with *KRAS* mutations to date. [6-9]

Prior to the exome result a diagnosis of Noonan syndrome had not been suspected as the patient did not have typical antenatal findings, facial features or significant cardiac or feeding abnormalities, and cloverleaf skull has not been reported previously in Noonan syndrome or related disorders. In light of the exome results the proband had an echocardiogram at almost 3 years of age; this was normal but she will continue cardiac surveillance. A coagulation screen has been normal but recommendations have been made to repeat it prior to any future surgery. Her development has been moderately delayed and her parents and community paediatricians have been given information about the spectrum of neurodevelopmental and behavioural phenotypes occurring in Noonan syndrome. In addition the finding of a *de novo* mutation accounting for her phenotype has facilitated accurate genetic counselling. A more complete account of this family will be published elsewhere.

Family 25: *EFNB1* mutation. The female proband (II-1 in Figure 1B), the first born child to healthy parents, with no relevant family history, was noted to have facial asymmetry at birth. Clinical assessment showed hypertelorism, orbital dystopia and ridging over the right coronal suture, but there were no additional dysmorphic features; in particular examination for signs of craniofrontonasal syndrome (CFNS; OMIM 304110; associated with grooving of the nasal tip, longitudinal splits of the finger- or toenails and cutaneous syndactyly) was negative (Figure 1B). A 3D-CT scan confirmed right coronal synostosis. She underwent a fronto-orbital advancement and remodelling (FOAR) procedure aged 15 months. On preoperative developmental assessment (Bayley Scales of Infant and Toddler Development, 3rd edition), composite scores ranged from 86 (language) to 100 (cognitive). Targeted sequencing of *FGFR2*, *FGFR3*, *TWIST1* and *TCF12* was normal.

Exome sequencing identified a heterozygous mutation (c.325C>T; p.Arg109Cys) in the X-linked *EFNB1* (ephrin-B1) gene, previously reported in a patient with CFNS. [10] Dideoxy-sequencing of the parents showed that the clinically unaffected father (I-1) was hemizygous for the same variant (Figure 1B). CFNS presents a paradoxical pattern of severity for an X-linked disorder, with

heterozygous females more severely affected than hemizygous males, who can be non-penetrant. [11] This phenomenon is accounted for by cellular interference. [12] This result predicts that 100% of female children of the father would be expected to exhibit CFNS and/or craniosynostosis, and the couple have elected to enrol in a programme of pre-implantation genetic diagnosis (PIGD), selecting only male embryos for uterine transfer.

Family 29: *STAT3* mutation. This boy (II-3 in Fig. 1C), the only child of unrelated parents of Sri Lankan ethnicity, presented to the craniofacial unit at 2 years of age. His parents had been concerned about his head shape at 6 months but he was not investigated until the age of 1 year 6 months. He had a history of recurrent ear infections and minor eczema behind his ears for which only emollients had been required. There was no relevant family history, except that his mother was recorded as having a large uterine fibroid. On examination he had mild midfacial hypoplasia, a short nose with a convex ridge, exorbitism and mild global development delay; 3D-CT scan demonstrated fusion of all of the cranial sutures with convexity of the closed anterior fontanelle, prominent ventricles and crowded basal cisterns. At the age of 2 years 2 months, ICP bolt insertion with 48 hour monitoring confirmed significantly raised ICP; ophthalmological assessment, which had previously been normal, showed progression to bilateral papilloedema. A sleep study did not show any obstructive sleep apnoea. A clinical diagnosis of Crouzon syndrome (OMIM 123500) was suspected but targeted sequencing of *FGFR2*, *FGFR3*, *TWIST1*, *TCF12* and *ERF* was normal. He underwent a posterior vault expansion with insertion of springs at the age of 28 months, with spring removal 8 months later. Formal dental review at 5 years of age found established primary dentition with a class III incisor relationship due to mid-facial hypoplasia.

Exome sequencing of the proband identified a heterozygous c.1915C>T (p.Pro639Ser) mutation in the SH2 domain encoded by *STAT3* (signal transducer and activator of transcription 3), which was previously reported in a case of Hyper-IgE/Jobs syndrome (HIES; OMIM 147060). [11] Dideoxy-sequencing of the parents showed that the mutation had arisen *de novo* (Figure 1C). Non-

immunological manifestations of HIES can include distinctive facial features, hyperextensibility of the joints, bone fractures and in rare cases, craniosynostosis. [13-15]

Upon report of this finding to the referring clinician it transpired that the proband had presented to his local hospital at the age of 3 years 3 months with an upper respiratory infection, progressing to severe necrotising pneumonia associated with a pulmonary abscess and pneumatocoele. Methicillin-resistant *Staphylococcus aureus* was initially isolated and subsequently he grew a *Stenotrophomonas maltophilia*. Despite intravenous antibiotics he developed a pneumothorax and bronchopleural fistula. Due to persistence of the fistula after two surgical resections, he was admitted to a specialist cardiothoracic unit at 3 years 7 months of age where he required a further thoracotomy with right lower lobe segmentectomy, resection of the 6th rib and an intercostal muscle pedicle to reinforce the repaired area. He was found to have a large secundum ASD for which he had a patch closure at 5 years of age.

In view of the identification of the *STAT3* mutation a detailed immunology assessment was undertaken. Of note, he had suffered a perforating otitis media with purulent discharge from which *Staphylococcus aureus* was isolated, and a single skin abscess on his neck at 3 years of age. As a baby he had had some cradle cap but no wider rash. There had been no significant gastrointestinal symptoms, sinusitis, tonsillitis or fractures. He had a molluscum contagiosum skin infection which was mild. His total IgE was 3091 kU/L (normal range 0-52). Other salient findings were a good response to tetanus vaccination but a very poor response to pneumococcal conjugate vaccination, and relatively normal lymphocyte subsets but with raised levels of γδ T cells indicating immune dysregulation. He was found to have EBV (IgE-VCA and IgM positive) and CMV (IgM negative, IgG positive) viraemias, of which the former has become chronic. Varicella zoster serology was negative and eosinophil counts have been consistently normal. He commenced prophylactic Azithromycin and Itraconazole and is currently awaiting a suitably matched donor for stem cell transplantation. Bone mineral density assessment was normal but he takes multivitamin supplements including vitamin D.

HIES is a multisystem immunodeficiency syndrome associated with a clinical triad of recurrent staphylococcal skin boils, cyst-forming pneumonias and extremely elevated IgE, usually manifesting in the first years of life. Typically it presents with a pustular rash in the newborn period which evolves into an eczematoid dermatitis. Other features include mucocutaneous candidiasis, osteopenia with recurrent fractures, scoliosis, craniosynsostosis which is said rarely to require surgery, [16-18] and failure to exfoliate the primary dentition. Vascular abnormalities include tortuosity of medium sized arteries and aneurysms. Gastro-intestinal features include gastro-oesophageal reflux disease, oesophageal dysmotility, and colonic diverticulae and perforations. There is also a predisposition to lymphoma. Management is directed at prevention and early aggressive treatment of pneumonia, abscesses and other infections using antibiotics, antifungal agents and skin antiseptics, treatment of eczema and optimisation of calcium and vitamin D intake. Surveillance for chest infections, scoliosis and retained primary dentition is indicated. A small number of patients have had stem cell transplantation with marked reduction in infections and significantly improved quality of life. Current recommendations in children with HIES associated with serious respiratory complications are to proceed to stem cell transplant once the child is clinically stabilised if there is a well matched donor available.

The proband had an atypical neonatal course and presented with a Crouzon-like facial appearance and pansynostosis prior to developing the more characteristic clinical features that might have pointed to a diagnosis of HIES. Demonstration of a *de novo STAT3* mutation accelerated referral for specialist immunological assessment and management allowing relatively early optimisation of treatment. As a result of the diagnosis closer dental and skeletal surveillance has been arranged. The findings have facilitated genetic counselling confirming that the parents have just a low germ-line mosaic recurrence risk for future pregnancies with prenatal testing available should they wish.

Family 37: *NTRK2* mutation. The female proband was the first born child to unrelated parents, with no relevant family history. Her birth weight was 3.2 kg (-0.57 SD) and she had a normal neonatal course. She presented with an asymmetric face at 12 months of age and left coronal synostosis was diagnosed on 3D-CT scan; there were no syndromic features and the head circumference at the age of 15 months was 47.0 cm (-0.28 SD). She underwent a FOAR procedure aged 17 months and has also required a strabismus correction. Formal testing at 17 months indicated low average attainment (composite scores on sub-tests 85-88, percentile rank 16-21). [19] Targeted sequencing of *FGFR2*, *FGFR3*, *TWIST1* and *TCF12*, MLPA of *TWIST1*, karyotype and array CGH were all normal.

By the age of 2 years 8 months she was noted to have episodes of temper tantrums and was exhibiting speech and language delay. On formal assessment at the age of 3 years 8 months, her standard scores for understanding and speaking language were 82 and 97, respectively. [20] Concerns about aggressive outbursts, ritualised behaviours, and language delay persisted at the age of 6 years and she required a school statement indicating moderate learning difficulties. In addition she was tall for her age with disproportionate weight gain, at the age of 6 years 8 months her height was 127.2 cm (+1.56 SD) and weight was 38.6 kg (+3.08 SD). Exome sequencing identified a heterozygous nonsense mutation (c.1330G>T; p.Gly444*) in *NTRK2*, encoding neurotrophic tyrosine receptor kinase, type 2, with a predicted loss of the intracellular tyrosine kinase domain. The proband's mother did not carry the mutation and the father was not available for analysis. Dominant mutations of *NTRK2* have previously been described in association with hyperphagic obesity associated with developmental delay (OBHD; OMIM #613886). [21]

The discovery of the *NTRK2* mutation prompted a further endocrinology assessment at the age of 7 years 6 months. Her height was 135.2 cm (+2.04 SD), weight 46.2 kg (+3.19 SD), and body mass index 25.3 kg/m^2 (+3.1 SD). She was noted to have a long-standing history of hyperphagia. The oral glucose tolerance test was normal; streak ovaries and uterus were evident on ultrasound scan. Management implications have included referral to a clinical psychologist and dietician to address

her eating behaviours, and regular monitoring for secondary complications including cardiovascular disease and diabetes. [13]

REFERENCES

1. Schepers D, Doyle AJ, Oswald G, Sparks E, Myers L, Willems PJ, Mansour S, Simpson MA, Frysira H, Maat-Kievit A, Van Minkelen R, Hoogeboom JM, Mortier GR, Titheradge H, Brueton L, Starr L, Stark Z, Ockeloen C, Lourenco CM, Blair E, Hobson E, Hurst J, Maystadt I, Destree A, Girisha KM, Miller M, Dietz HC, Loeys B, Van Laer L. The SMAD-binding domain of SKI: a hotspot for de novo mutations causing Shprintzen-Goldberg syndrome. European journal of human genetics : EJHG. 2015;23(2):224-8.
2. Kempers M, Salemink S, Spruijt L, Marcelis C, Loeys B. Unusual presentations of thoracic aortic aneurysm in children. The Fourth Cardiff Cardiovascular Genetics Symposium. 2013.
3. Jacquinet A, Verloes A, Callewaert B, Coremans C, Coucke P, de Paepe A, Kornak U, Lebrun F, Lombet J, Pierard GE, Robinson PN, Symoens S, Van Maldergem L, Debray FG. Neonatal progeroid variant of Marfan syndrome with congenital lipodystrophy results from mutations at the 3' end of FBN1 gene. Eur J Med Genet. 2014;57(5):230-4.
4. Kosaki K, Takahashi D, Udaka T, Kosaki R, Matsumoto M, Ibe S, Isobe T, Tanaka Y, Takahashi T. Molecular pathology of Shprintzen-Goldberg syndrome. Am J Med Genet A. 2006;140(1):104-8; author reply 9-10.
5. Sood S, Eldadah ZA, Krause WL, McIntosh I, Dietz HC. Mutation in fibrillin-1 and the Marfanoid-craniosynostosis (Shprintzen-Goldberg) syndrome. Nat Genet. 1996;12(2):209-11.
6. Schubbert S, Zenker M, Rowe SL, Boll S, Klein C, Bollag G, van der Burgt I, Musante L, Kalscheuer V, Wehner LE, Nguyen H, West B, Zhang KY, Sistermans E, Rauch A, Niemeyer CM, Shannon K, Kratz CP. Germline KRAS mutations cause Noonan syndrome. Nat Genet. 2006;38(3):331-6.
7. Brasil AS, Malaquias AC, Kim CA, Krieger JE, Jorge AA, Pereira AC, Bertola DR. KRAS gene mutations in Noonan syndrome familial cases cluster in the vicinity of the switch II region of the G-domain: report of another family with metopic craniosynostosis. Am J Med Genet A. 2012;158A(5):1178-84.
8. Addissie YA, Kotecha U, Hart RA, Martinez AF, Kruszka P, Muenke M. Craniosynostosis and Noonan syndrome with KRAS mutations: Expanding the phenotype with a case report and review of the literature. Am J Med Genet A. 2015;167a(11):2657-63.
9. Kratz CP, Zampino G, Kriek M, Kant SG, Leoni C, Pantaleoni F, Oudesluys-Murphy AM, Di Rocco C, Kloska SP, Tartaglia M, Zenker M. Craniosynostosis in patients with Noonan syndrome caused by germline KRAS mutations. Am J Med Genet A. 2009;149a(5):1036-40.
10. Wallis D, Lacbawan F, Jain M, Der Kaloustian VM, Steiner CE, Moeschler JB, Losken HW, Kaitila, II, Cantrell S, Proud VK, Carey JC, Day DW, Lev D, Teebi AS, Robinson LK, Hoyme HE, Al-Torki N, Siegel-Bartelt J, Mulliken JB, Robin NH, Saavedra D, Zackai EH, Muenke M. Additional EFNB1 mutations in craniofrontonasal syndrome. Am J Med Genet A. 2008;146A(15):2008-12.
11. Ozylmaz B, Gezdirici A, Ozen M, Kalenderer O. Report of a family with craniofrontonasal syndrome. Clin Dysmorphol. 2015;24(2):79-83.
12. Wieacker P, Wieland I. Clinical and genetic aspects of craniofrontonasal syndrome: towards resolving a genetic paradox. Molecular genetics and metabolism. 2005;86(1-2):110-6.
13. Kokkoris P, Pi-Sunyer FX. Obesity and endocrine disease. Endocrinol Metab Clin North Am. 2003;32(4):895-914.
14. Nieminen P, Morgan NV, Fenwick AL, Parmanen S, Veistinen L, Mikkola ML, van der Spek PJ, Giraud A, Judd L, Arte S, Brueton LA, Wall SA, Mathijssen IM, Maher ER, Wilkie AO, Kreiborg S,

Thesleff I. Inactivation of IL11 signaling causes craniosynostosis, delayed tooth eruption, and supernumerary teeth. Am J Hum Genet. 2011;89(1):67-81.
15. Richards S, Aziz N, Bale S, Bick D, Das S, Gastier-Foster J, Grody WW, Hegde M, Lyon E, Spector E, Voelkerding K, Rehm HL. Standards and guidelines for the interpretation of sequence variants: a joint consensus recommendation of the American College of Medical Genetics and Genomics and the Association for Molecular Pathology. Genet Med. 2015;17(5):405-24.
16. Gahr M, Muller W, Allgeier B, Speer CP. A boy with recurrent infections, impaired PMN-chemotaxis, increased IgE concentrations and cranial synostosis--a variant of the hyper-IgE syndrome? Helv Paediat Acta. 1987;42(2-3):185-90.
17. Hoger PH, Boltshauser E, Hitzig WH. Craniosynostosis in hyper-IgE-syndrome. Eur J Pediatr. 1985;144(4):414-7.
18. Smithwick EM, Finelt M, Pahwa S, Good RA, Naspitz CK, Mendes NF, Kopersztyk S, Spira TJ, Nahmias AJ. Cranial synostosis in Job's syndrome. Lancet. 1978;1(8068):826.
19. Michalec D. Bayley Scales of Infant Development: Third Edition. In: Goldstein S, Naglieri JA, editors. Encyclopedia of Child Behavior and Development. Boston, MA: Springer US; 2011. p. 215.
20. Zimmerman IL, Pond RE, Steiner VG. PLS-5UK: Preschool Language Scale Fifth Edition ed: Pearson; 2014.
21. Yeo GS, Connie Hung CC, Rochford J, Keogh J, Gray J, Sivaramakrishnan S, O'Rahilly S, Farooqi IS. A de novo mutation affecting human TrkB associated with severe obesity and developmental delay. Nat Neurosci. 2004;7(11):1187-9.
